# Nicotinamide Phosphoribosyltransferase Acetylation Mediating Muscle Dysfunction Contributes to Sleep Apnoea in Obesity

**DOI:** 10.1002/jcsm.13693

**Published:** 2025-02-03

**Authors:** Liu Zhang, Ya Ru Yan, Shi Qi Li, Ying Ni Lin, Yi Wang, Yu Qing Wang, Ning Li, Fang Ying Lu, Xian Wen Sun, Li Yue Zhang, Jian Ping Zhou, Yong Jie Ding, Qing Yun Li

**Affiliations:** ^1^ Department of Respiratory and Critical Care Medicine, Ruijin Hospital Shanghai Jiao Tong University School of Medicine Shanghai China; ^2^ Institute of Respiratory Diseases Shanghai Jiao Tong University School of Medicine Shanghai China; ^3^ Department of Internal Medicine, Montefiore Medical Center Albeit Einstein College of Medicine New York New York USA

**Keywords:** muscle, nicotinamide phosphoribosyltransferase, obesity, obstructive sleep apnoea

## Abstract

**Background:**

Obstructive sleep apnoea (OSA) occurs frequently among individuals with obesity, which is attributed to upper airway muscle dysfunction. Muscle function is regulated by the dynamic balance of the nicotinamide adenine dinucleotide (NAD+) and its reduced form (NADH), which is controlled by the enzyme nicotinamide phosphoribosyltransferase (NAMPT). Elevated NAMPT levels have been found in individuals with obesity. However, the role of NAMPT in obesity‐induced muscle impairment has not been fully clarified.

**Methods:**

A total of 110 participants (70 moderate‐to‐severe OSA vs. 40 mild or no OSA) underwent electrical impedance mammography and polysomnography. C57BL/6J mice with high‐fat diet‐induced obesity (DIO) and control group were utilized for their characterizations, which included forced running wheel tests, glucose tolerance tests, haematoxylin and eosin staining, immunostaining, magnetic resonance imaging, whole‐body plethysmography, electromyographic techniques, western blot, NAMPT enzymatic activity assays and NAD+/NADH ratio measurements.

**Results:**

Patients with moderate–severe OSA have a significant decrease in lean mass percentage of upper airway muscles compared with those in controls (*p* < 0.01). In vivo, a high‐fat diet reduced the levels of NAD‐dependent deacetylase sirtuin‐1 (SIRT1) (*p* < 0.01), which plays a crucial role in the deacetylation of NAMPT. The reduction in SIRT1‐mediated NAMPT deacetylation (*p* < 0.001) resulted in decreased NAMPT activity (*p* < 0.01), leading to a decrease in NAD+/NADH ratio (*p* < 0.05) and decreased the myosin heavy chain isoform (MyHC) I level (*p* < 0.05), thereby affecting the effectiveness of upper airway muscle and ultimately leading to upper airway collapse (101.0 vs. 81.7 pixels, *p* = 0.02). The introduction of estradiol mitigated high‐fat diet‐induced muscle dysfunction by enhancing expression of SIRT1 and inhibiting the acetylation of NAMPT, reducing upper airway collapse (81.7 vs. 96.7 pixels, *p* = 0.06).

**Conclusions:**

These findings highlight the crucial role of SIRT1‐mediated NAMPT deacetylation on obesity‐induced muscle dysfunction, suggesting targeting NAMPT has the potential to reverse the obesity induced muscle dysfunction and provide effective treatment options for OSA.

## 1 Introduction

Obstructive sleep apnoea (OSA) is characterized by recurrent periods of upper airway obstruction and intermittent hypoxia [1]. Approximately 936 million people worldwide suffer from OSA, of which 425 million have moderate‐to‐severe forms [2]. OSA increases the risk of conditions such as hypertension, stroke and diabetes, and even neurocognitive sequelae, imposing a substantial economic burden on healthcare resources [3, 4]. However, effective therapeutics for OSA remain scarce [5]. One of the major risk factors contributing to OSA is obesity. Even a modest 10% increase in body weight can amplify the risk by 6‐fold and the severity by 32% [6]. Obesity typically exacerbates OSA by causing the accumulation of local fat in the upper airways and affecting the structure and function of the upper airway muscles (UAM) [7]. However, the mechanism by which obesity affects the muscles has yet to be fully elucidated.

Impaired energy metabolism and mitochondrial dysfunction are critical for obesity‐induced muscle dysfunction [8], which can result from imbalance of nicotinamide adenine dinucleotide (NAD+) and its reduced form, NAD+ hydrogen (NADH) [9]. NAD+ repletion provides protection form metabolic diseases and mitochondrial dysfunction induced by diet or ageing [10, 11]. There are three major independent pathways for NAD+ synthesis, of which the salvage pathway is predominantly used by mammalian cells to regenerate degraded NAD+ locally, with nicotinamide phosphoribosyltransferase (NAMPT) serving as the rate‐limiting component of this pathway [12].

Expression and activity of NAMPT are involved in metabolism and function of muscle [13]. Circulating NAMPT levels are associated with insulin resistance and nutritional status among the elderly [14]. Moreover, NAMPT levels were recognized as a key regulator of chronic inflammatory, which is related to sarcopenia [15]. In addition, NAMPT regulates the synthesis and degradation of muscle proteins, affecting muscle mass [16].

Typically, fluctuations in NAD+ levels correlate with those in NAMPT. For instance, concurrent reductions in both NAD+ and NAMPT have been observed during ageing and in myopathies [9, 17]. However, in obesity, a decrease in NAD+/NADH ratio is associated with an increase in NAMPT levels, paradoxically [17] (Data S1). The underlying mechanism for this phenomenon remains unexplored. Yoon et al. found that the acetylation level of NAMPT significantly affects its secretion and enzymatic activity, which was controlled by sirtuin‐1 (a NAD+‐dependent protein deacetylase, SIRT1) [18]. Therefore, we hypothesized that, in obesity, the balance between NAD+/NADH ratio and the levels of NAMPT is regulated by the enzyme NAMPT through acetylation.

Thus, we aimed to investigate whether high‐fat diet impaired UAM dysfunction may be because of the NAMPT deacetylation. Mice with high‐fat diet‐induced obesity (DIO) were used to investigate this hypothesis, in which sleep disordered breathing is similarly to human disease [19]. Our findings establish the role of NAMPT acetylation level in UAM injury and shed light on potential mechanisms driving obesity‐related declines in UAM function.

## 2 Methods

### 2.1 Key Resources Table

**Table:** 

Reagent or resource	Source	Identifier
Antibodies		
MyHC I	Abcam	ab11083
MyHC IIa	CST	3403
MyHC IIb	CST	3404
SIRT1	ABclonal	A0230
NAMPT	ABclonal	A0256
Acetylated‐lysine	CST	9441
Normal rabbit lgG	CST	2729
Tubulin	CST	2146
HSP90	Abmart	T55548
Critical commercial assays		
NAMPT Activity Assay Kit (colorimetric)	Abcam	ab221819
NAD/NADH Assay Kit	Abcam	ab65348
Experimental models		
Cell line: C2C12	Cell Bank of the Chinese Academy of Sciences	
Mouse: C57BL/6J		Vital River
SOFTWARE		
Prism 8		GraphPad
Other		
SRT1720	MCE	HY‐15145
Estradiol	MCE	HY‐B0141

### 2.2 Human Subjects

**FIGURE 1:**
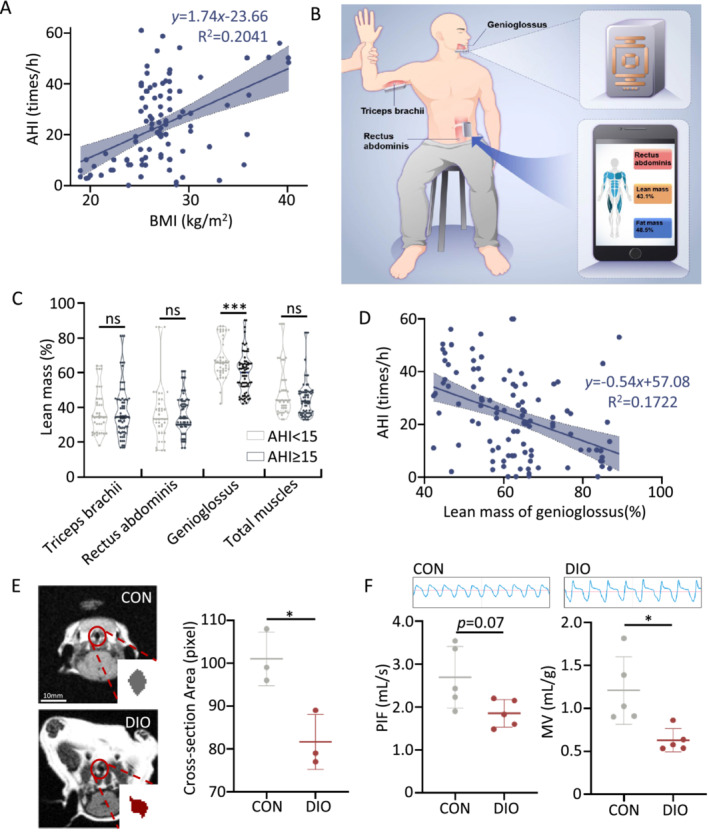
Structure and function of upper airway muscles in obesity population and diet‐induced obesity (DIO) mouse model. (A) Scatter plot shows a positive correlation between apnoea–hypopnea index (AHI) and body mass index (BMI) in a sample size of 110 participants, indicating that patients with obesity have more severe sleep apnoea. (B) Schematic diagram of electrical impedance mammography (EIM) to measure lean mass and fat mass of three muscles. (C) The percentage change in lean mass in patients with obstructive sleep apnoea (OSA) compared with that in controls, showing that patients with OSA have lower genioglossus lean mass. (D) Scatter plot shows a negative association between the AHI and genioglossus lean mass, indicating that the lean mass of the genioglossus muscle may contribute to OSA pathogenesis. (E) Representative axial MRI images of the upper airway (left). Calculation of cross‐sectional area in upper airway area (right). (F) The DIO groups have airflow obstruction compared with the control group. Airflow represents the raw signal via whole‐body plethysmography in anaesthetised mice (up). Calculation of maximum peak inspiratory flow (PIF) in mice (*n* = 5) (left); Calculation of minute ventilation (MV) in mice. (*n* = 5) (right). Error bars represent SD. *p < 0.05 and ***p < 0.001 by Student's *t*‐test.

This is a cross‐sectional study in Figures 1 and 6A,B. Participants with suspected OSA (snoring, witnessed apnoeas or daytime sleepiness) were enrolled consecutively in the study from Ruijin Hospital, Shanghai Jiao Tong University School of Medicine between March 2021 and February 2022. Exclusion criteria were as follows: (1) patients showing complications with severe respiratory diseases, such as severe chronic obstructive pulmonary disease, interstitial lung disease or acute asthma; (2) patients showing complications with serious cardiovascular diseases such as acute myocardial infarction, acute heart failure or chronic congestive heart failure (grades III and IV); (3) patients with mental illnesses who could not cooperate with the examination; (4) patients who receiving noninvasive positive pressure ventilation therapy and (5) patients who might have other sleep disorders under clinical evaluation. Age, sex and body mass index (BMI) were recorded in Table S1. All participants underwent electrical impedance myography (EIM) and polysomnography (PSG). Genioglossus represents UAM, whereas triceps brachii and rectus abdominis represents large muscle groups. The other investigation is also a cross‐sectional study that consecutively enrolled a total of 49 healthy subjects without any chronic disease or OSA in Figure S2A.

The study was approved by the Ethics Committee of Shanghai Jiao Tong University School of Medicine at Ruijin Hospital (Protocol #2018‐107). Written informed consent was obtained from all participants.

### 2.3 Animal Models

Animal experiments were approved by the Ethics Committee of Ruijin Hospital (Shanghai, China). C57BL/6J was purchased from the Weitong Lihua Company (Beijing, China). Male C57BL/6J mice (5‐week‐old) were randomly divided into the control and diet‐induced obesity (DIO) group. DIO mice were fed with high‐fat diet (Research diets D12492) with 60% calories derived from fat (lard) and 20% from sucrose for 16 weeks. Since the eighth week, the DIO mice were injected intraperitoneally with SIRT1 activator, SRT1720 (50 mg/kg/day) or estradiol (10 mg/kg/day) every other day for 8 weeks to construct DIO + SRT or DIO + E2 model. The same volume of solvent was administered in a similar manner. All mice were utilized for their characterizations including forced running wheel tests, glucose tolerance tests, haematoxylin and eosin staining, immunostaining, magnetic resonance imaging (MRI), whole‐body plethysmography, electromyographic (EMG) study, western blot, nicotinamide phosphoribosyltransferase (NAMPT) enzymatic activity assays and NAD+/NADH ratio measurements. All mice were housed at 22°C on a 12/12‐h light/dark cycle in a group of four to five. Cages and bedding were changed once per week. The health status of mice was monitored daily.

### 2.4 Cell Culture

C2C12 cell lines were cultured in Dulbecco's modified Eagle medium (DMEM) supplemented with 10% foetal bovine serum (FBS, Gibco) and 1% antibiotics. C2C12 were differentiated by replacing the culture medium with differentiation media, including DMEM, 2% horse serum (Sigma) and 1% antibiotics for 5 days.

### 2.5 PSG

All participants underwent overnight PSG. No coffee, tea or sedative hypnotics were taken before sleep. The PSG monitoring included electrooculography, electroencephalography, electrocardiography, chin electromyography (EMG), measurements of thoracal and abdominal movements, airflow pressure, thermistor readings and oxygen saturation (Alice 5, Philips Respironics, USA). OSA was defined by apnoea–hypopnea index (AHI) ≥ 15 events/h according to the American Academy of Sleep Medicine 2007 criteria.

### 2.6 Electrical Impedance Mammography (EIM)

The muscle and fat masses of the total, triceps brachii, abdominal and genioglossus muscles were measured via Skulpt Chisel. All measurements were taken with the muscle in a relaxed state from the top of the muscle bulk at predefined anatomical locations (see schematic Figure 1B). The muscle masses and fat content were calculated by EIM phase value (degree) at 50 kHz and the ratio of EIM phase value at 50 kHz/200 kHz. Investigators were blinded to these data at the time of collection.

### 2.7 Fasting Blood Glucose and Glucose Tolerance Test

Blood samples were collected from the orbital venous plexus and were determined by Yuwell fasting glucometer (Yuyue, Shandong, China). After 16 h of fasting, glucose (2 g/kg body weight) was administered by intraperitoneal injection. Blood glucose levels were measured before glucose administration (0 min) and after 15, 30, 60 and 120 min.

### 2.8 Haematoxylin and Eosin Staining

The gastrocnemius and tongue tissue from a subset of animals (*n* = 4–5) were fixed in buffered 4% formalin followed by formalin fixation. Serial muscle cross‐sections of 5 μm were collected using an automatic microtome. The samples were immediately fixed in microscope slides and stained with haematoxylin–eosin for microscopy.

### 2.9 Immunofluorescence

The gastrocnemius and tongue tissue from a subset of animals (*n* = 4–5) were fixed in buffered 4% formalin followed by formalin fixation. Serial muscle cross‐sections of 5 μm were collected using an automatic microtome. The samples were immediately fixed in microscope slides and stained with MyHCI (ab11083), MyHCIIb (#3403) and 4′,6‐diamidino‐2‐phenylindole (DAPI).

### 2.10 Forced Running Wheel

The forced running wheel experiments were carried out with the mice rotarod fatigue meter (YLS‐10B). All mice were trained to adapt to the meter. After acclimatization, all mice were subjected to the formal experiment with a 9 m/min rotation speed until exhaustion or for 30 min. The criterion of exhaustion was defined as the point at which mice stayed on the electric shocker without trying to resume running.

### 2.11 Body Composition

The body composition of live mice was examined via MRI (QMR23‐060H‐I). The measurement was repeated three times, and the average value was taken.

### 2.12 MRI Imaging

Mice were anaesthetised by inhalation of isoflurane in oxygen in an induction box. The mouse head was fixed in place with a tooth bar in a cradle. The mouse was inserted head‐first into the MRI scanner (Niu Mai Instruments, Suzhou, M7). A single midline sagittal image was acquired to identify the junction between the hard palate and soft palate, which was used as a landmark for defining subsequent axial scans.

The imaging protocols were both coronal and transverse scans. For transverse scans, the image was acquired with repetition time = 500 ms, effective echo time = 12 ms, field‐of‐view = 60 × 100 mm, number of slices = 20 and slice thickness = 1 mm. To obtain the contrast, grey‐level images are processed with pseudo‐colour, as shown in Figures S1D and S4I, with red indicating body fat.

Additional coronal scans were performed from the junction of the hard and soft palates. Respiration was monitored using a pneumatic sensor. The image was acquired with repetition time = 547.3 ms, effective echo time = 15 ms, field‐of‐view = 60 × 60 mm, number of slices = 20 and slice thickness = 1 mm. Data were transferred to Image J (NIH, Bethesda, MD, USA) and measured the maximum cross‐sectional area of the upper airway. All cross‐sectional areas were measured using the same thresholding subroutine and protocol by an observer (L.Z.).

### 2.13 Whole‐Body Plethysmograph (WBP)

Mice were anaesthetised using sodium pentobarbital and were placed in the whole‐body plethysmography for testing. After the respiratory waveform became stable, the test lasted 10 min for each of the mice. The stable waveform > 5 min was selected for analysis.

### 2.14 Genioglossus EMG

In anaesthetised supine animals, a small midline incision was made along the submentum. Two electrodes were inserted in the genioglossus muscle. The EMG signal was amplified and filtered between 30 and 1000 Hz. After the signal became stable, the frequency was analysed.

### 2.15 Western Blot

The genioglossus lysates and C2C12 cells were lysed with whole cell lysis buffer at 100°C for 15 min and then centrifuged at 100 × *g* for 10 min. The supernatants were collected for subsequent analyses. Protein lysates were resolved by 10%–12% SDS‐PAGE and transferred to a polyvinylidene difluoride (PVDF) membrane. Membranes were incubated overnight with primary antibodies at 4°C. Secondary antibody incubation was conducted at room temperature for 1 h. All antibody details are found in Section 2.1. Protein signals were visualized using the Immobilon Western Kit (Millipore, USA). Data were quantified from at least three independent assays.

### 2.16 Co‐Immunoprecipitation (Co‐IP)

Co‐IP was performed using the Protein A/G Magnetic Beads IP Kit (Biolinkedin) according to the manufacturer's instructions. Protein A/G Magnetic Beads were bound with antibodies and incubated overnight at 4°C with shaking. Thereafter, cell lysates were incubated together with antibody‐bound magnetic beads overnight at 4°C rotating. After washing the beads five times, the above complex was resuspended in a lysis buffer. Consequently, the lysis buffer was used for western blotting.

### 2.17 Quantitative Real‐Time PCR

Total cellular RNA was extracted using Trizol (Thermo Fisher Scientific) following the manufacturer's instructions. SYBR Premix Ex Taq (Takara, Shiga, Japan) was used to quantitative real‐time PCR on an ABI 7500 system. Relative expression levels were determined based on Ct values and normalized to tubulin.

### 2.18 NAMPT Enzymatic Activity

The NAMPT enzymatic activity was determined from the C2C12 cell and genioglossus, using the NAMPT Activity Assay Kit (Abcam, ab221819). The operations were conducted strictly according to the instructions. The optical density (OD) values were obtained at 450 nm, and the activity was assessed.

### 2.19 NAD+/NADH Ratio

NAD+ and NADH were quantified using the NAD+/NADH Assay Kit (Abcam, ab65348). Cells (2 × 106) were treated with 400‐μL extraction buffer using three freeze/thaw cycles. Extracted samples (50 μL) were heated to 60°C for 20 min to detect NADH. Extracted samples (50 μL) were used to detect NAD+. Thereafter, the wells were mixed with cycling buffer, enzyme mix and NADH developer. The absorbance of OD 450 nm was detected with a spectrophotometer. The amounts of NADH and NAD+ were calculated using the standard curve in the kit.

### 2.20 Acetylation Mass Spectrometry

C2C12 cell samples were lysed, and proteins were extracted and digested. Acetylated peptides were enriched with pretreated Anti‐Ac‐K antibody beads (PTMScan Acetyl–Lysine Motif [Ac‐K] Kit, CST). Liquid chromatography (LC)–mass spectrometry (MS)/MS analysis was performed on a timsTOF Pro mass spectrometer (Bruker) that was coupled to nanoelute (Bruker Daltonics). The MS raw data for each sample were combined and searched using the MaxQuant software for identification and quantitation analysis. Data analysis was performed using the Zhongke data analysis platform (https://bio‐cloud.aptbiotech.com).

### 2.21 Statistical Analysis

Data are expressed as the mean ± standard deviation (mean ± SD). Statistical tests used for analysis were two‐tailed Student's *t*‐test and ANOVA. Statistical analyses were performed using SPSS 24.0 (SPSS Inc., Chicago, IL, USA) and GraphPad Prism 8.0.2 (GraphPad Software, Inc., USA). Statistical significance was set at *p* < 0.05. For all experiments, all stated replicates are biological replicates.

## 3 Results

### 3.1 Upper Airway Muscles Composition Affect the Severity of Obesity‐Related OSA

A total of 110 participants (70 moderate‐to‐severe OSA, defined by an apnoea–hypopnea index (AHI) ≥ 15 events/h, and 40 mild or non‐OSA with AHI < 15 events/h, Table S1) were enrolled in the project. The results indicated a positive correlation between BMI and AHI (Figure 1A). Then, body composition analysis (BCA) was measured on the genioglossus (represents UAM), triceps brachii and rectus abdominis (represents large muscle groups) (Figure 1B). A significant decrease in lean mass percentage was observed in the genioglossus of patients with moderate–severe OSA compared with controls (*p* < 0.01; Figure 1C), whereas no significant differences were observed in the muscle masses of the triceps brachii and rectus abdominis (*p* > 0.05; Figure 1C). In terms of fat mass, a reduction of fat content was observed in the genioglossus and triceps brachii, but not in the rectus abdominis (Figure S1A). Additionally, a negative correlation between the lean mass of genioglossus and AHI was found (Figure 1D). These findings indicate that modifications in the UAM may contribute to the development of OSA, with an emphasis on the genioglossus muscle.

### 3.2 High‐Fat Diets Enhance the Narrowing of the Upper Airway in Mice

To investigate if obesity contributes to the development of OSA by affecting UAM, C57BL/6 mice were fed with high‐fat diet to induce diet‐induced obesity (DIO) mouse model. Compared with the control group (CON), the DIO mice exhibited an increase in body weight (Figure S1B) and impaired glucose tolerance (Figure S1C) (*p* < 0.05). Meanwhile, the DIO group displayed a decrease in lean mass and an increase in body fat content (Figure S1D–F), and a significant decrease in forced wheel‐running distance (Figure S1G), indicating compromised exercise capacity.

Numerous studies have focused on skeletal muscle in the limbs, such as the anterior tibialis muscle and the gastrocnemius muscle [20]; we also analysed the changes in gastrocnemius muscle (Figure S1H,I). However, several differences exist between the limb muscles and UAM. To investigate the impact of a high‐fat diet on the structure and function of the UAM, we used cross‐sectional T1 weighted MRI to examine the cross‐sectional area of upper airway structure of mice. The results indicate that the cross‐sectional area of the upper airway in DIO mice were narrower than the control group (101.0 vs. 81.7 pixels, *p* = 0.02) (Figure 1E). Additionally, the respiratory function was assessed using whole‐body plethysmography (Figure 1F). The maximum peak inspiratory flow (PIF) tended to decrease in DIO mice, but with no statistical difference (*p* = 0.07; Figure 1F) compared with control group. Meanwhile, DIO mice also have a significant decrease in minute ventilation (MV) (Figure 1F), which was negatively correlated with the severity of upper airway obstruction. These results confirmed that obesity‐induced narrowing of the upper airway, thereby impacting its structural integrity and impairing respiratory function.

### 3.3 High‐Fat Diet Increases Responsiveness and Decreases Effectiveness of the Genioglossus, Contributing to Upper Airway Collapsibility

**FIGURE 2:**
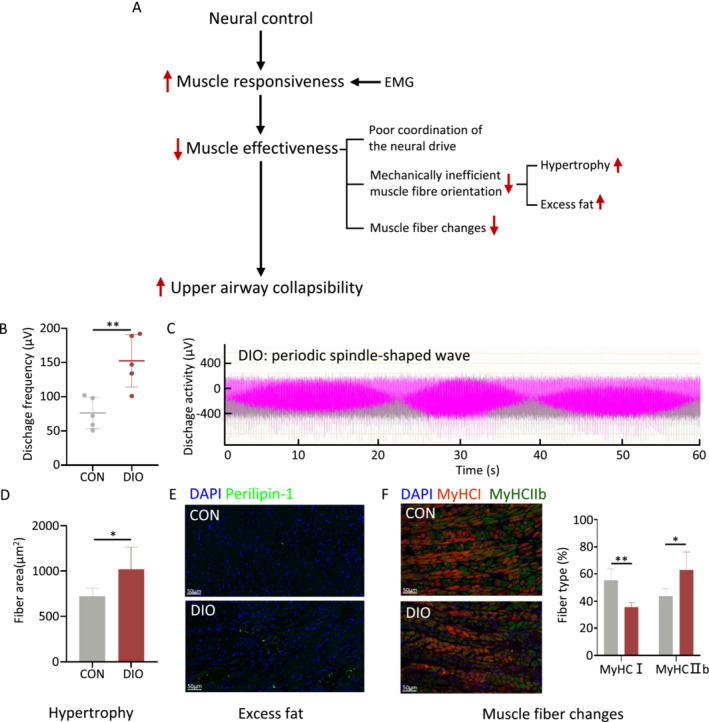
High‐fat diet impairs muscle effectiveness, but not responsiveness in the diet‐induced obesity (DIO) group. (A) The process and relevant mechanism of skeletal muscle contraction, including neural control, muscle responsiveness and muscle effectiveness. Red arrows indicate the findings from this study in the DIO group. Muscle responsiveness is evaluated through electromyographic (EMG) techniques. (B) Discharge frequency of the genioglossus EMG study (CON *n* = 5, DIO *n* = 5). (C) Periodic changes in genioglossus EMG study in the DIO group at 1 min. Muscle effectiveness includes mechanically inefficient muscle fibre orientation—caused by muscle hypertrophy (D) or excess fat (E)—or alterations in muscle fibre type (F). (D) Quantification of myofibre cross‐sectional area of genioglossus. (E) Representative immunohistochemical staining for perilipin in the genioglossus. Green = perilipin; blue = 4′,6‐diamidino‐2‐phenylindole (DAPI). (F) Representative immunohistochemical staining for myosin heavy chain (MyHC) I and MyHCIIb in the genioglossus (left). Quantitative data for the immunofluorescence intensities of MyHC1 and MyHCIIb (right). (*n* = 5). Green = MyHCI‐positive myofibres; red = MyHCIIb; blue = DAPI. Error bars represent SD. *p < 0.05, **p < 0.01, ***p < 0.001 by Student's *t*‐test.

The upper airway lacks osseous support structures and instead depends on the contraction of UAM to sustain openness, among which the genioglossus holds a crucial role [21]. The process of muscle contraction proceeds through three fundamental steps: neural control, muscle responsiveness and muscle effectiveness (Figure 2A) [22]. Muscle responsiveness is the ability of UAM to increase their activity when challenged with respiratory stimuli (i.e., CO_2_), which can be assessed by the EMG activity of the UAM [23]. Our study demonstrated that the DIO group exhibited higher EMG amplitudes and frequencies in the genioglossus muscle compared with the control group (Figure 2B), indicating elevated responsiveness. Specifically, EMG recording obtained from the DIO group revealed the presence of periodic spindle‐shaped waves (Figure 2C, Video S1), indicating the recurring fluctuation in tension of the genioglossus muscle. This pattern is indicative of repetitive collapse and obstruction of the upper airway in DIO mice during sleep.

Muscle effectiveness refers to the ability of the UAM to translate the neural drive received into airway dilation and increased airflow [23], which is regulated by alterations in muscle fibre type and other factors, such as mechanically inefficient muscle fibre orientation (caused by excess fat or muscle hypertrophy) [23]. Our study demonstrated a larger cross‐sectional area of muscle fibres of the genioglossus muscle in the DIO group (Figure 2D). Additionally, lipid droplet surface protein perilipin levels were found to be increased (Figure 2E), indicating an increase in intermuscular fat in the DIO group. Immunofluorescence staining further revealed changes in the proportion of muscle fibres, specifically a decrease in the slow fibre MyHCI and an increase in the fast fibre MyHCIIb (Figure 2F). These alterations suggested that the DIO group had muscle hypertrophy, an increase in intermuscular fat and a decrease in the proportion of slow fibres, resulting in a reduction in muscle effectiveness [23].

These observations indicate that a high‐fat diet plays a role in augmenting the responsiveness of the genioglossus while diminishing its effectiveness (Figure 2A), ultimately leading to the collapse of the upper airway.

### 3.4 NAMPT Acetylation Disrupts the Balance of NAD+/NADH, Resulting in Decreased MyHCI, Contributing to Decreased Muscle Effectiveness

**FIGURE 3:**
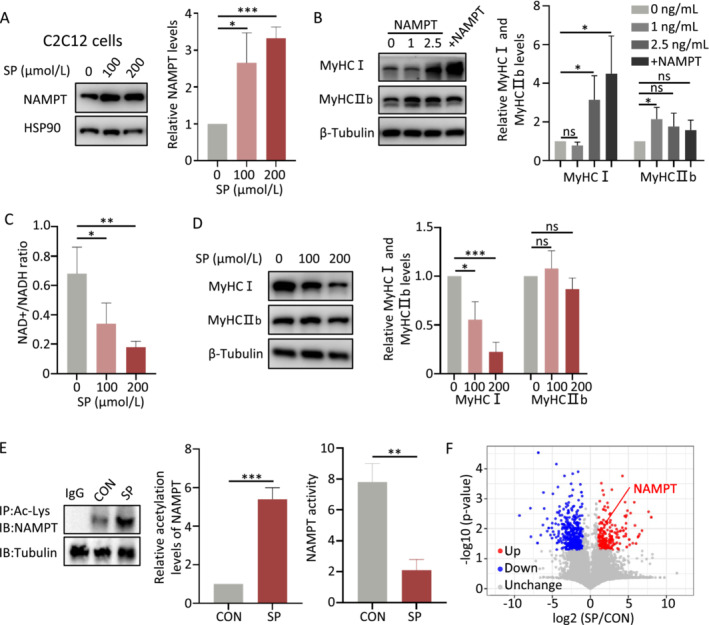
High‐fat diet decreases MyHC I level, related to the enzymatic activity and acetylation level of NAMPT. (A) Protein levels of NAMPT in C2C12 cells treated with different sodium palmitate (SP) concentrations (*n* = 3). (B) Protein levels of myosin heavy chain (MyHC) I and MyHCIIb in C2C12 cells treated with different NAMPT concentrations or overexpressed NAMPT (+NAMPT) (*n* = 3). (C) The NAD+/NADH ratio in C2C12 cells treated with different SP concentrations. (*n* = 3). (D) Protein levels of MyHCI and MyHCIIb in C2C12 cells treated with different SP concentrations. (*n* = 3). (E) Acetylated NAMPT protein levels (left) and enzymatic activity of NAMPT (right) in SP‐treated C2C12 cells (*n* = 3). (F) Volcano plots of quantitative acetylated mass spectrometry (MS) results. IP, immunoprecipitation; IB, immunoblot. Error bars represent SD. *p < 0.05, **p < 0.01, ***p < 0.001 by Student's *t*‐test.

NAD+ is one of the most important components in energy metabolism of skeletal muscle [9]. We further explored the NAMPT and NAD/NADH ratio in human, animal models and C2C12 cells. From our human study, we found that serum NAMPT levels were positively correlated with BMI in 49 healthy to the healthy group (Figure S2A), elevated NAMPT levels were also found in OSA compared with the healthy group (Figure S2B). In animal models, elevation of NAMPT in genioglossus cells was observed in the DIO group in vivo (Figure S2C), as well as in the serum of DIO mice compared with the control group (Figure S2D). To further investigate the impact of high‐fat diets, we conducted in vitro experiments using C2C12 cells, which were treated with sodium palmitate (SP) to mimic the high‐fat diet model [23]. Increased NAMPT levels were also observed in C2C12 cells treated with SP in vitro (Figure 3A). Under exercise, or ageing and various myopathies, NAMPT levels correlate with NAD+/NADH ratio positively [24] (Data S2). Similar results were obtained in the present study that addition or overexpression of NAMPT results in C2C12 cells increased NAD+/NADH ratio (Figure S2E) and MyHCI protein expression (Figure 3B). However, in obesity, we observed an opposite pattern. In C2C12 cells treated with increased concentration of SP, we found a reduction in intracellular NAD+/NADH ratio paradoxically (Figure 3C). Similarly, in the DIO mice model, we found a decrease in NAD+/NADH ratio in the setting of elevated NAMPT levels in the genioglossus (Figure S2F). We further examined the muscle fibre changes in vitro, the results revealed a notable decrease in MyHCI protein levels following SP treatment, whereas no significant change was observed in MyHCIIb (Figure 3D). The mRNA levels of both MyHCI and MyHCIIb exhibited a similar pattern (Figure S2G).

Next, we sought to explore the reasons behind the paradoxical changes of NAMPT and NAD+/NADH ratio levels in obesity. We found that treatment with SP increased acetylation levels of NAMPT in C2C12 cells (Figure 3E). Correspondingly, the enzymatic activity of NAMPT decreased compared with that in the control cells (Figure 3E). To further substantiate the findings, the acetylation levels of all proteins in both SP and control groups were assessed through acetylation mass spectrometry. The acetylation level of NAMPT, as indicated by the volcano plots, emerged as one of the significantly upregulated proteins (Figure 3F). Additionally, the specific acetylation site of NAMPT was identified as K469. These outcomes imply that a high‐fat diet resulted in elevated levels of acetylated NAMPT, consequently leading to reduced enzymatic activity and impaired synthesis of NAD+ (Figure 8).

### 3.5 High‐Fat Diet Inhibits SIRT1‐Mediated NAMPT Deacetylation

**FIGURE 4:**
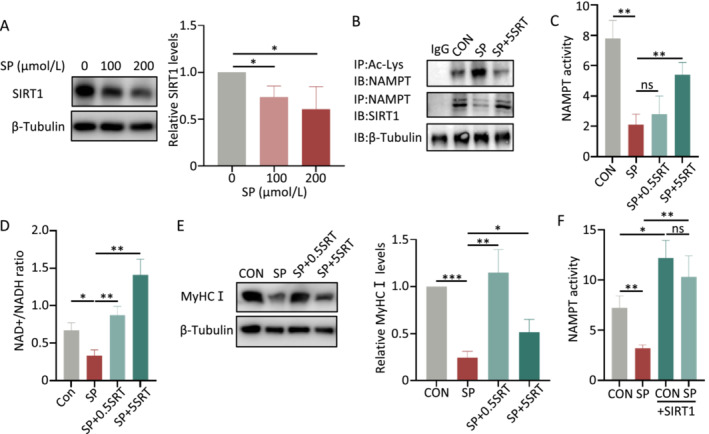
SIRT1 agonists increases levels of MyHCI by reducing the acetylated NAMPT levels and enhancing its enzymatic activity in vitro. (A) Protein levels of SIRT1 in C2C12 cells treated with different sodium palmitate (SP) concentrations (*n* = 3). (B) Immunoprecipitation (IP) assay of acetylated nicotinamide phosphoribosyltransferase (NAMPT) protein levels and co‐immunoprecipitation (Co‐IP) assay of endogenous NAMPT and SIRT1 in C2C12 cells treated with SP and SRT1720 (SRT) (*n* = 3). (C) The enzymatic activity of NAMPT, (D) intracellular NAD+/NADH ratio and (E) protein levels of MyHCI in C2C12 cells treated with SP and SRT (0.5, 5 mg/mL) (*n* = 3). (F) Enzymatic activity of NAMPT in SP‐treated C2C12 cells overexpressing SIRT1 (+SIRT1) (*n* = 3). IP, Immunoprecipitation; IB, immunoblot. Error bars represent SD. *p < 0.05, **p < 0.01, ***p < 0.001 by Student's *t*‐test.

Sirtuin 1 (SIRT1) is an NAD+‐dependent protein deacetylase, which increases NAMPT deacetylation levels and increases its enzymatic activity [18]. We observed a significant downregulation of the SIRT1 protein (Figure 4A) and mRNA expression levels (Figure S3A) in C2C12 cell lines treated with SP. Furthermore, SIRT1 agonist SRT1720 (SRT) decreased the NAMPT acetylation levels (Figures 4B and S3B) and enhanced NAMPT activity (Figure 4C), elevated the NAD+/NADH ratio (Figure 4D) and upregulated the expression of MyHCI protein (Figure 4E). Using a lentiviral vector, we generated a stable SIRT1 overexpressed C2C12 cell lines. We found an enhanced NAMPT activity (Figure 4F) and increase in the MyHCI level (Figure S3C) in these cells. Meanwhile, immunofluorescence analysis revealed extensive intracellular co‐localization of SIRT1 and NAMPT (Figure S3D), and Co‐IP assay provided evidence that SIRT1 and NAMPT have a direct interaction (Figure 4B).

**FIGURE 5:**
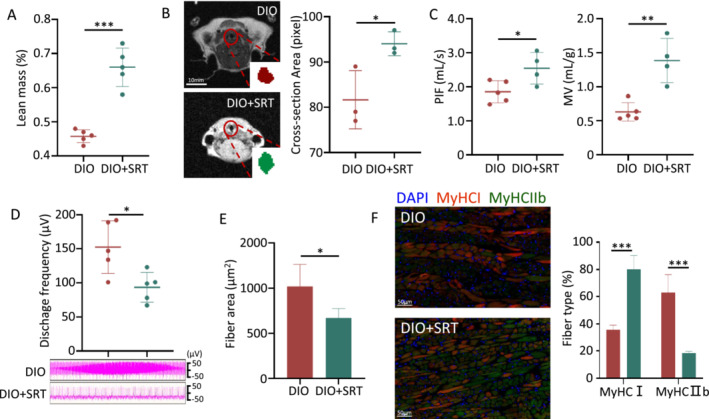
SIRT1 agonists improve upper airway structure and function in DIO mouse group. (A) MRI measurement of lean mass percentage in live, awake mice (DIO *n* = 5, DIO + SRT *n* = 5). (B) Representative axial MRI of the upper airway (left) and calculation of cross‐sectional morphology (right) of upper airway. (C) Calculation of maximum peak inspiratory flow (PIF) (left) and minute ventilation (MV) (right) via whole‐body plethysmography (DIO *n* = 5, DIO + SRT *n* = 4). (D) Discharge frequency of the genioglossus electromyographic (EMG) recordings in the DIO and DIO + SRT groups. (DIO *n* = 5, DIO + SRT *n* = 4). (E) Cross‐sectional area of the genioglossus fibres in the DIO and DIO + SRT groups. (F) Representative immunohistochemical staining for MyHCI and MyHCIIb in the genioglossus (left). Green = MyHCI‐positive myofibres; red = MyHCIIb; blue = DAPI. Quantitative data for the immunofluorescence intensities of MyHCI and MyHCIIb (right) (DIO *n* = 5, DIO + SRT *n* = 4). Error bars represent SD. *p < 0.05, **p < 0.01, ***p < 0.001 by Student's *t*‐test.

In vivo, DIO + SRT mice (the DIO mice were injected with SIRT1 activator, SRT1720) exhibited notable reductions in body weight (Figure S3E), improved glucose tolerance (Figure S3F) and forced wheel‐running distance (Figure S3G), compared with the DIO group. BCA revealed a significant decrease in fat mass (Figure S3H) and an increase in lean mass (Figure 5A). A reduction in the cross‐sectional area of muscle fibres (Figure S3I) and an increase in the proportion of MyHCI (Figure S3J) in the DIO + SRT group were observed in the gastrocnemius.

We found an increase in upper airway cross‐sectional area (94.0 vs. 81.7 pixels, *p* = 0.04) (Figure 5B) and improved respiratory function (MV and PIF) (Figure 5C) in the DIO + SRT mice, indicating that SRT1720 ameliorated upper airway narrowing effectively. Based on EMG studies of the genioglossus, the DIO + SRT group exhibited a decrease in frequency and absence of periodicity than that in the DIO group, indicating decreased UAM responsiveness (Figure 5D). Although the DIO + SRT group exhibited a reduction in muscle fibre cross‐sectional area (Figure 5E), a decrease in perilipin levels (Figure S3K) and an increase in MyHCI proportions (Figure 5F) suggest improved UAM effectiveness. These findings demonstrate SIRT1 agonist ameliorated UAM dysfunction induced by a high‐fat diet (Figure 8).

### 3.6 Estradiol Rescues UAM Dysfunction Through the SIRT1–NAMPT Deacetylation Pathway

**FIGURE 6:**
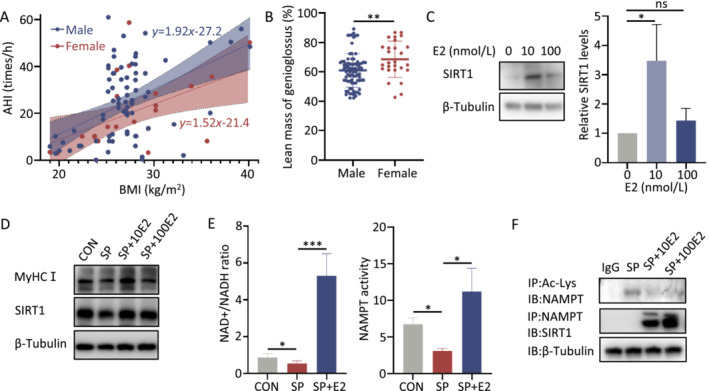
Estradiol (E2) increases levels of MyHCI by reducing the acetylated NAMPT levels and enhancing its enzymatic activity in vitro*.* (A) Linear relationship between the apnoea–hypopnea index (AHI) and body mass index (BMI), discriminated by sex (*n* = 110), which revealed a lower correlation between BMI and AHI in the female group than those in the male. (B) Lean mass of genioglossus in male (*n* = 83) and female subjects (*n* = 27). (C) SIRT1 protein levels in C2C12 cells treated with estradiol (E2) (0, 10, 100 nmol/L) (*n* = 3). (D) Myosin heavy chain (MyHC) I and SIRT1 protein levels, (E) intracellular NAD+/NADH ratio (left) and enzymatic activity of NAMPT (right) in C2C12 cells treated with sodium palmitate (SP) and E2 (*n* = 4). (F) Immunoprecipitation (IP) assay of acetylated NAMPT protein levels and co‐immunoprecipitation (Co‐IP) of endogenous NAMPT and SIRT1 in C2C12 cells treated with SP, SP + SRT1720 and SP + E2 (estradiol) (*n* = 3). IP, immunoprecipitation; IB, immunoblot. Error bars represent SD. *p < 0.05, **p < 0.01, ***p < 0.001 by Student's *t*‐test.

In human study, subgroup analyses revealed a lower correlation between BMI and AHI in the female group than those in the male (Figure 6A), indicating that obesity exerts less influence on the development of OSA in females. Meanwhile, females exhibited a greater muscle percentage of genioglossus compared with those in males (Figure 6B), suggesting that upper airway muscle plays a role in the disparities between sexes.

17β‐estradiol (E2) is a possible candidate responsible for the sex differences [25, 26]. We further explored whether estradiol can alleviate obesity‐induced muscle dysfunction mediated by the SIRT1–NAMPT pathway. At a physiological concentration of 10 μmol/L, estradiol exhibited an increase in the SIRT1 protein level in C2C12 cells (Figure 6C), without significantly affecting the mRNA level (Figure S4A), although it has minimal impact on the MyHCI protein level (Figure S4B) or the NAD+/NADH ratio (Figure S4C). Estradiol has been shown to have protective effects on upper airway muscles under various stressors, such as a high‐fat diet and intermittent hypoxia [27] (Data S3). In the present study, we found that estradiol resulted in increased protein levels of MyHCI and SIRT1 in SP‐treated C2C12 cell lines, as well as an elevated NAD+/NADH ratio compared with the SP group (Figures 6D,E and S4D). The deacetylation level and enzymatic activity of NAMPT exhibited significant trends, similar to the SIRT1 protein level (Figures 6E,F and S4E). The Co‐IP assay confirmed the increased co‐precipitation of SIRT1 with NAMPT (Figure 6F).

In vivo, the DIO + E2 mice (estradiol supplementation to DIO male mice) exhibited a decrease in body weight (Figure S4F), but no discernible difference was observed in forced wheel‐running distance (*p* = 0.08, Figure S4G) and glucose tolerance (Figure S4H). Analysis of body composition revealed a significant reduction in fat mass (Figure S4I,J) and an increase in lean mass (Figure S4I,K). No statistically significant differences were observed in the cross‐sectional area of muscle fibres (Figure S4L) and muscle fibre proportion (Figure S4M) in the gastrocnemius.

**FIGURE 7:**
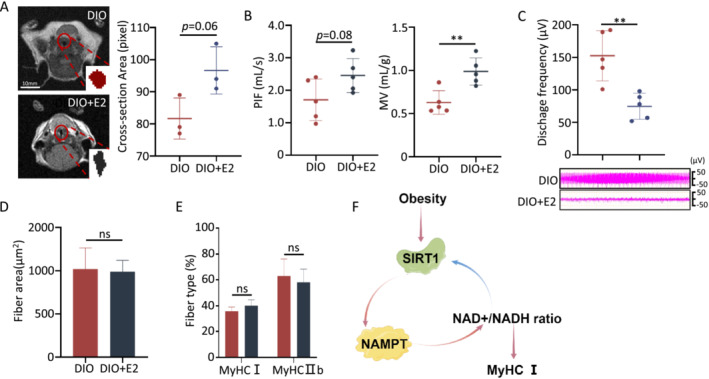
Estradiol (E2) improve upper airway structure and function in DIO mouse. (A) Representative axial MRI of upper airway (left) and calculation of cross‐sectional area (right) in upper airway area. (B) Airflow represents the raw signal via whole‐body plethysmography in anaesthetised mice. Calculation of maximum peak inspiratory flow (PIF) (left) and minute ventilation (MV) (*n* = 5) (right). (C) Genioglossus electromyographic (EMG) recordings of the DIO and DIO + E2 groups (*n* = 5) (up). Discharge frequency of the genioglossus EMG study (down). (D) Cross‐sectional area of the genioglossus fibres in the DIO and DIO + E2 groups. (E) Quantitative data for the immunofluorescence intensities of MyHCI and MyHCIIb of the genioglossus in the DIO and DIO + E2 groups (*n* = 5). (F) Schematic diagram of the positive feedback loop of SIRT1/NAMPT/NAD+/SIRT1 pathway. We found that a high‐fat diet lowers the expression of SIRT1, leading to increased NAMPT acetylation levels which in return reduced its activity and declined NAD+/NADH ratio (red arrows). Combined with that SIRT1 is an NAD+‐dependent deacetylase (blue arrow), this forms a positive feedback involving SIRT1/NAMPT/NAD+/SIRT1 pathway. Error bars represent SD. *p < 0.05, **p < 0.01, ***p < 0.001 by Student's *t*‐test.

In the upper airway, DIO + E2 mice group showed the trend of larger cross‐sectional area of the upper airway compared with control group, but no significantly (96.7 vs. 81.7 pixels, *p* = 0.06, Figure 7A). There is also a tendency towards increased PIF (Figure 7B, *p* = 0.08), but no difference, and a significantly increase in MV (Figure 7B, *p* < 0.01), indicating E2 has protective effects in DIO male mice against narrowing of the upper airway. Moreover, in the DIO + E2 group, the discharge frequency of EMG decreased significantly, and the presence of periodic spindle wave signs was absent (Figure 7C). No differences in cross‐sectional area (Figure 7D), perilipin levels (Figure S4N) and the proportions of MyHCI of UAM (Figure 7E) were found between the DIO and DIO + E2 groups. These findings provide partial evidence regarding the protective effects of E2 on the impaired UAM caused by a high‐fat diet.

## 4 Discussion

OSA is distinguished by the repetitive collapse of the upper airway during sleep. Unlike the lower airway, the upper airway lacks osseous support structures and instead depends on the contraction of UAM to sustain openness. Over 20 skeletal muscles are involved in the process of upper airway dilation, with the genioglossus muscle being of utmost importance as it significantly contributes to the development of OSA [21]. Previous studies concerning the effects of obesity on skeletal muscles have predominantly focused on limb muscles such as the gastrocnemius and extensor digitorum longus. However, evaluating the genioglossus muscles presents unique challenges because of their location. Various techniques have been utilized in clinical settings to evaluate muscle composition, such as dual‐energy X‐ray absorption, MRI, bioelectrical impedance analysis and EIM [28]. EIM has garnered attention because of its noninvasive characteristics and convenient assessment of localized muscle composition [29, 30]. By using EIM, we found a notable disparity in lean mass of the genioglossus among patients with moderate‐to‐severe OSA compared with the control group, thereby emphasizing its potential role in assessing UAM as a noninvasive method. However, several limitations should be mentioned. First, patients with OSA are usually be older and have more comorbidities than controls, which may be confounding factors for results. Second, the cross‐sectional design and small sample size of the study makes it difficult to establish the causal relationship between variables.

The contraction of genioglossus occurs in three sequential steps, namely, neural control, muscle responsiveness and muscle effectiveness [22]. In this study, EMG results indicated a significant increase in muscle responsiveness of the genioglossus in DIO mice. However, decreased muscle responsiveness is believed to contribute OSA pathogeneses [31, 32]. This may be because of compensatory mechanisms, and more investigations are needed in the future. Meanwhile, we confirmed that a high‐fat diet led to mechanically inefficient muscle fibre orientation (including muscle hypertrophy and excess fat) and muscle fibre changes, ultimately leading to reduced muscle effectiveness and resulting in upper airway collapse. Previous studies have primarily focused on increasing neural control or muscle responsiveness through the administration of 5‐hydroxytryptamine 3 antagonists (e.g., ondansetron) and norepinephrine reuptake inhibitors (e.g., protriptyline or desipramine) [22]. It is recommended that future research should focus on the development of drug treatments that specifically target muscle effectiveness.

NAMPT plays a crucial role in NAD+ production, as generally, the levels of NAD+ increase with NAMPT [24]. However, in obesity, we found that increased NAMPT levels are not followed by increased NAD+ levels. This contradiction could be attributed to post‐translational modification, such as acetylation, resulting in decreased enzymatic activity of NAMPT, as Yoon et al. have reported [18]. We found that a high‐fat diet leads to increased NAMPT acetylation levels, reducing its enzymatic activity, thus decreasing NAD+ synthesis, and causing NAD+/NADH imbalance. We named this phenomenon as “NAMPT resistance.” Additionally, our study revealed that obesity decreased SIRT1 levels, which leads to increased NAMPT acetylation level and decreased NAD+/NADH ratio. Combined with that SIRT1 is an NAD+‐dependent deacetylase, which forms positive feedback involving SIRT1/NAMPT/NAD+/SIRT1 (Figure 7F). Intervention targeting NAMPT has the potential to break the vicious cycle of SIRT1/NAMPT/NAD+/SIRT1 and reverse the clinical course of obesity induced OSA.

Additionally, male sex is a significant risk factor for OSA. Males exhibit a greater tendency towards increased airway obstruction per change in BMI than females [33] (Data S4), suggesting the impact of obesity on the upper airway collapse may differ between sexes. Animal studies have suggested that male mice are more susceptible to the negative metabolic consequences of high‐fat diets than female mice [34, 35]. Sex‐related differences in NAD+ homeostasis and related interventions also have been documented [36, 37]. Yoshino et al. conducted a study wherein NAD+ precursor supplements were administered to a cohort of 25 postmenopausal overweight or obese women with prediabetes, which revealed a notable enhancement in muscle insulin sensitivity and signalling [38]. In contrast, other studies about NAD+ precursor supplements did not yield positive results on glucose tolerance in males [39, 40]. Additionally, the animal models found that increased NAD+ by NAMPT overexpression exhibited greater prominence in female mice [36]. These findings highlight the considerable variations in the effects of NAD+ supplementation based on sex, suggesting a potential association between NAMPT and estradiol. In this study, we revealed that estradiol could influence NAMPT acetylation by modulating SIRT1 levels, subsequently impacting its enzyme activity and altering the skeletal muscle structure and function. Therefore, estradiol could mitigate the effects of obesity on UAM damage by enhancing SIRT1‐mediated NAMPT deacetylation, providing insights into sex differences in obesity‐associated OSA.

**FIGURE 8:**
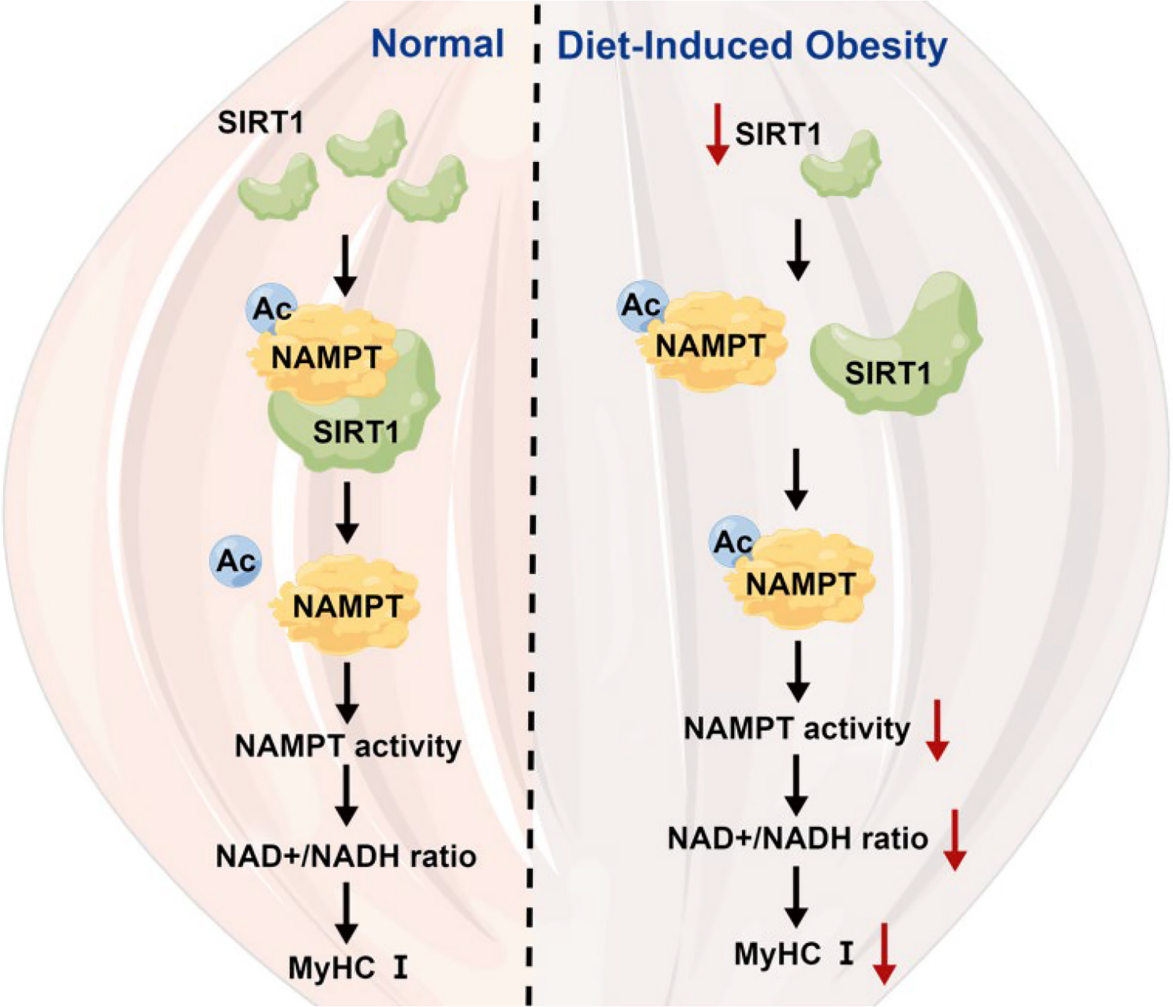
Mechanism diagram of high‐fat diet‐induced upper airway collapsibility. High‐fat diet reduces the SIRT1 levels responsible for deacetylating nicotinamide phosphoribosyltransferase (NAMPT). This reduction in SIRT1‐mediated NAMPT deacetylation leads to decreased NAMPT activity, resulting in a diminished NAD+/NADH ratio. In vivo, this disruption in NAD+ homeostasis decreased MyHCI, ultimately leading to upper airway collapse. Red arrows indicate the findings from this study in the diet‐induced obesity (DIO) group. NAMPT‐Ac, the acetylation level of NAMPT.

Altogether, our project revealed that the high‐fat diet leads to a reduction of NAD and an imbalance of NAD/NADH+ level by decreasing SIRT1‐mediated NAMPT deacetylation. SIRT1 agonist decreased NAMPT acetylation level and enhanced NAMPT activity in vitro and in vivo (Figure 8). These results imply the potential role of targeting SIRT1‐mediated NAMPT acetylation for the treatment of OSA associated with obesity. Moreover, the 17β‐estradiol alleviates the muscle abnormality by the SIRT1–NAMPT deacetylation pathway, providing insights into sex differences in obesity‐associated OSA.

## Author Contributions

Q.Y.L. conceived and supervised the entire study. L.Z. and F.Y.L. designed the experiments. L.Z. and Y.R.Y. performed most of the experiments related to Figures 1, 2, 3; S.Q.L., Y.N.L. and Y.W. performed experiments related to Figures 4, 5, 6. Y.Q.W. revised the manuscript; X.W.S. and L.Y. Z. wrote the manuscript. N.L., J.P.Z. and Y.J.D. collected clinical data and analysed the data.

## Conflicts of Interest

The authors declare no conflicts of interest.

## Supporting information


**Figure S1.** The effect of a high‐fat diet on mice and obstructive sleep apnea (OSA) patients (related to Figure 1). (A) Percentage change in fat mass of patients with OSA and healthy controls. (B) Weight and (C) blood glucose of the control (CON) and DIO groups.(D) Whole‐body T1 weighted magnetic resonance imaging (MRI) of representative mice in the CON and diet‐induced obesity (DIO)groups, with red and blue indicating body fat and skeletal muscle, respectively. MRI measurement of (E) lean mass percentage and (F) total body fat percentage in live, awake mice (*n* = 5). (G) Changes in forced wheel‐running distance in mice (*n* = 5).(H) Immunohistochemistry staining results of the gastrocnemius (left) and cross‐sectional area of the gastrocnemius fibres (right).(I) Representative immunohistochemical staining for MyHCI and MyHCIIb in the gastrocnemius (left). Green = myosin heavy chain (MyHC) I‐positive myofibers; red = MyHCIIb; blue = DAPI. Quantitative data for the immunofluorescence intensities of MyHCI and MyHCIIb (right). Error bars represent SD. *p < 0.05, **p < 0.01, ***p < 0.001 by student’s t test.
**Figure S2.** High‐fat diet increases NAMPT protein levels, but decreases NAD+/NADH ratio (related to Figure 3). (A) Linear relationship between serum NAMPT levels and body mass index (BMI) in healthy male subjects (*n* = 49), indicating that patients with obesity have higher serum NAMPT levels. (B) Levels of serum NAMPT in patients with obstructive sleep apnea (OSA) and healthy controls. (CON *n* = 14, OSA *n* = 12). (C) Intracellular nicotinamide phosphoribosyltransferase (NAMPT) levels of the genioglossus in the CON and DIO groups. (*n* = 3). (D) Levels of serum NAMPT in the CON and diet‐induced obesity (DIO) groups. (CON *n* = 6, DIO *n* = 11). (E) Intracellular NAD+/NADH ratio in C2C12 cells treated with different NAMPT concentrations or overexpressed NAMPT (+NAMPT) (*n* = 3). (F) The NAD+/NADH ratio in the CON and DIO groups. (CON *n* = 5, DIO n = 5). (G) Messenger RNA (mRNA) levels of myosin heavy chain (MyHC) I and MyHCIIb in C2C12 cells treated with different sodium palmitate (SP) concentrations. (*n* = 3). Error bars represent SD. *p < 0.05, **p < 0.01, ***p < 0.001 by student’s t test.
**Figure S3.** SIRT1 agonists ameliorate weight, blood glucose and structure of the gastrocnemius in DIO group (related to Figure 4 and 5). (A) Messenger RNA (mRNA) levels of SIRT1 in C2C12 cells treated with different sodium palmitate (SP) concentrations (*n* = 3). (B) Densitometric quantification of the relative band intensity in Figure 4B (*n* = 3). (C) Protein levels of MyHCI in C2C12 cells overexpressing SIRT1 (+SIRT1) and the control group (n = 3). (D) Representative immunohistochemical staining for NAMPT and SIRT1 in the genioglossus. Green = NAMPT‐positive myofibers; red = SIRT1; blue = DAPI. (E) Weight and (F) blood glucose of the CON, DIO and DIO + SRT groups. (G) Changes in forced wheel‐running distance (DIO *n* = 5, DIO + SRT *n* = 4). (H) MRI measurement of total body fat percentage in live, awake mice (DIO n = 5, DIO + SRT n = 5). (I) Immunohistochemistry staining results of the gastrocnemius (left) and cross‐sectional area of the gastrocnemius fibres (right). (J) Representative immunohistochemical staining for MyHCI and MyHCIIb in the gastrocnemius (left). Green = myosin heavy chain (MyHC) I‐positive myofibers; red = MyHCIIb; blue = DAPI. Quantitative data for the immunofluorescence intensities of MyHCI and MyHCIIb (right). (K) Representative immunohistochemical staining for perilipin in the genioglossus. Green = perilipin; blue = 4′,6‐diamidino‐2‐phenylindole (DAPI) (DIO *n* = 5, DIO + SRT *n* = 4). Error bars represent SD. *p < 0.05, **p < 0.01, ***p < 0.001 by student’s t test.
**Figure S4.** Estradiol ameliorate weight, blood glucose and structure of the gastrocnemius in DIO group via increasing SIRT1 level (related to Figure 6 and 7). (A) Relative SIRT1 mRNA levels, (B) MyHCI protein levels and (C) intracellular NAD+/NADH ratio in C2C12 cells treated with estradiol. (D) Densitometric quantification of the relative band intensity in Figure 6D (*n* = 3). (E) Densitometric quantification of the relative band intensity in Figure 6F (n = 3). (F) Weight and (H) blood glucose of the CON, DIO and DIO + E2 groups. (G) Changes in forced wheel‐running distance (DIO *n* = 5, DIO + E2 n = 5). (I) Pseudo‐colour photon image of magnetic resonance imaging (MRI) in the diet‐induced obesity (DIO) and DIO + E2 groups, in which red and blue indicate body fat and skeletal muscle, respectively. MRI measurement of (J) total body fat percentage and (K) lean mass percentage in live, awake mice. (L) Immunohistochemistry staining results of the gastrocnemius (left) and cross‐sectional area of the gastrocnemius fibres (right). (M) Representative immunohistochemical staining for MyHCI and MyHCIIb in the gastrocnemius (left). Green = myosin heavy chain (MyHC) I‐positive myofibers; red = MyHCIIb; blue = DAPI. Quantitative data for the immunofluorescence intensities of MyHCI and MyHCIIb (right). (N) Representative immunohistochemical staining for perilipin in the genioglossus. Green = perilipin; blue = 4′,6‐diamidino‐2‐phenylindole (DAPI) (*n* = 3). Error bars represent SD. *p < 0.05, **p < 0.01, ***p < 0.001 by student’s t test.

**Table S1.** Baseline characteristics and body composition of the study population.

**Video S1.** Representative periodic spindle‐shaped wave of EMG in DIO group. The video showed the presence of periodic spindle‐shaped waves of EMG recording in DIO group, which signified the recurring fluctuation in tension of the genioglossus muscle. This pattern is indicative of repetitive collapse and obstruction of the upper airway during sleep.

**Data S1.** Supplementary References.
