# Supplementary material for: Nicotinamide Phosphoribosyltransferase Acetylation Mediating Muscle Dysfunction Contributes to Sleep Apnoea in Obesity
Source: J Cachexia Sarcopenia Muscle. 2025 Feb 3;16(1):e13693. doi: 10.1002/jcsm.13693 (PMC11790607; doi:10.1002/jcsm.13693)
Supplement: Supplementary file 1 — Figure S1. The effect of a high‐fat diet on mice and obstructive sleep apnea (OSA) patients (related to Figure 1). (A) Percentage change in fat mass of patients with OSA and healthy controls. (B) Weight and (C) blood glucose of the control (CON) and DIO groups.(D) Whole‐body T1 weighted magnetic resonance imaging (MRI) of representative mice in the CON and diet‐induced obesity (DIO)groups, with red and blue indicating body fat and skeletal muscle, respectively. MRI measurement of (E) lean mass percentage and (F) total body fat percentage in live, awake mice (n = 5). (G) Changes in forced wheel‐running distance in mice (n = 5).(H) Immunohistochemistry staining results of the gastrocnemius (left) and cross‐sectional area of the gastrocnemius fibres (right).(I) Representative immunohistochemical staining for MyHCI and MyHCIIb in the gastrocnemius (left). Green = myosin heavy chain (MyHC) I‐positive myofibers; red = MyHCIIb; blue = DAPI. Quantitative data for the immunofluorescence intensities of MyHCI and MyHCIIb (right). Error bars represent SD. *p < 0.05, **p < 0.01, ***p < 0.001 by student’s t test. Figure S2. High‐fat diet increases NAMPT protein levels, but decreases NAD+/NADH ratio (related to Figure 3). (A) Linear relationship between serum NAMPT levels and body mass index (BMI) in healthy male subjects (n = 49), indicating that patients with obesity have higher serum NAMPT levels. (B) Levels of serum NAMPT in patients with obstructive sleep apnea (OSA) and healthy controls. (CON n = 14, OSA n = 12). (C) Intracellular nicotinamide phosphoribosyltransferase (NAMPT) levels of the genioglossus in the CON and DIO groups. (n = 3). (D) Levels of serum NAMPT in the CON and diet‐induced obesity (DIO) groups. (CON n = 6, DIO n = 11). (E) Intracellular NAD+/NADH ratio in C2C12 cells treated with different NAMPT concentrations or overexpressed NAMPT (+NAMPT) (n = 3). (F) The NAD+/NADH ratio in the CON and DIO groups. (CON n = 5, DIO n = 5). (G) Messenger RNA (mRNA) levels [file JCSM-16-e13693-s004.pdf]

Nicotinamide Phosphoribosyltransferase Acetylation Mediating Muscle Dysfunction Contributes to Sleep Apnea in Obesity

Journal of Cachexia, Sarcopenia and Muscle

Liu Zhang, Ya Ru Yan, Shi Qi Li, Ying Ni Lin, Yi Wang,Yu Qing Wang, Ning Li, Fang Ying Lu, Xian Wen Sun, Li Yue Zhang, Jian Ping Zhou, Yong Jie Ding, Qing Yun Li

Correspondence: Qing Yun Li, M.D, Ph.D.
Department of Respiratory and Critical Care Medicine, Ruijin Hospital, Shanghai Jiao Tong University School of Medicine, Shanghai 200025, China
Email: liqingyun68@hotmail.com

Figure S1

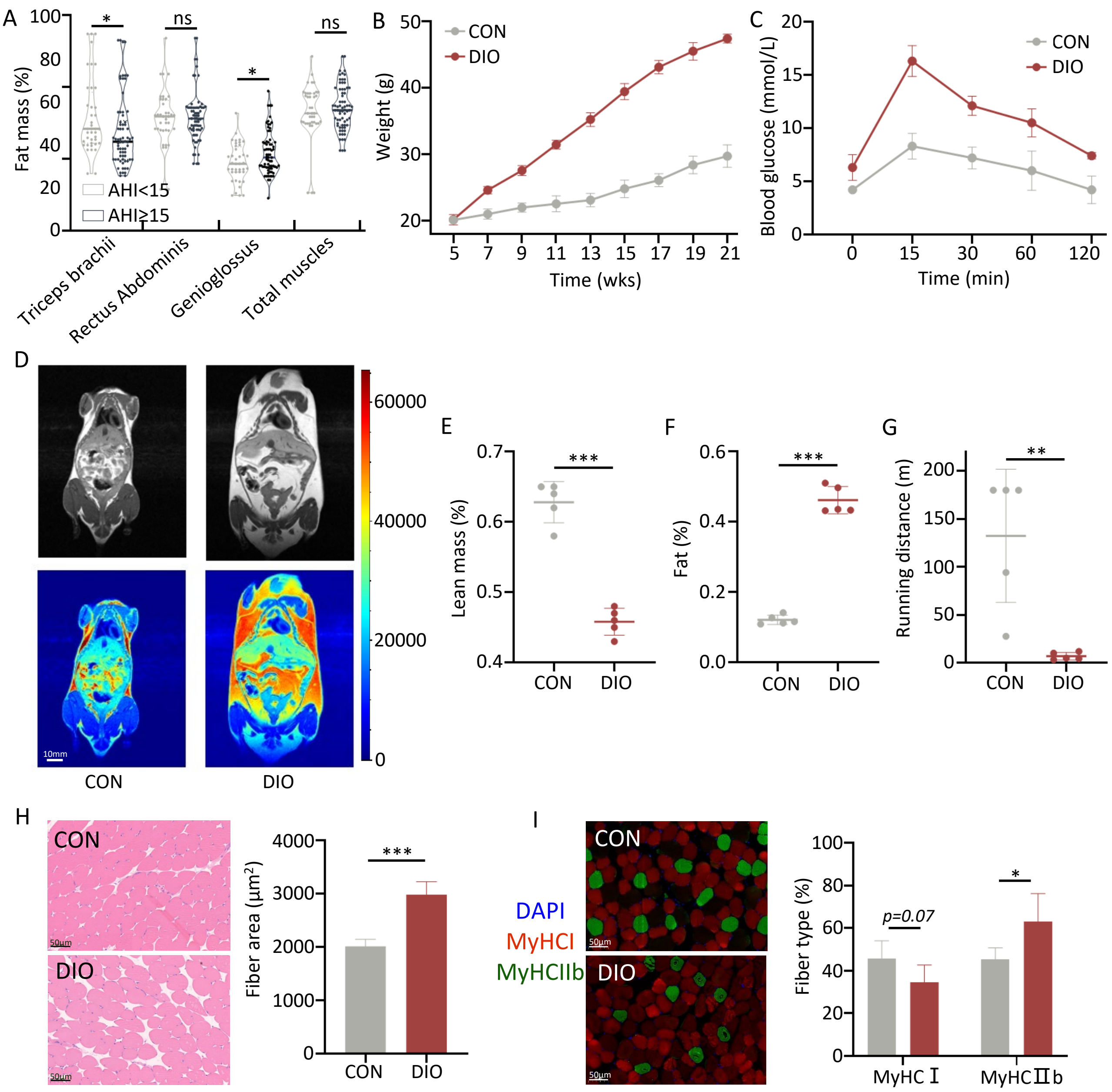
A
Fat mass (%)
*
ns
*
ns
AHI<15
AHI≥15
Triceps brachii
Rectus Abdominis
Genioglossus
Total muscles
B
Weight (g)
CON
DIO
Time (wks)
C
Blood glucose (mmol/L)
CON
DIO
Time (min)
D
CON
DIO
10mm
E
Lean mass (%)
***
F
Fat (%)
***
G
Running distance (m)
**
H
CON
DIO
50μm
Fiber area (μm²)
***
I
DAPI
MyHCI
MyHCIIb
CON
DIO
50μm
Fiber type (%)
p=0.07
*
MyHC Ⅰ
MyHC Ⅱb

Figure S2

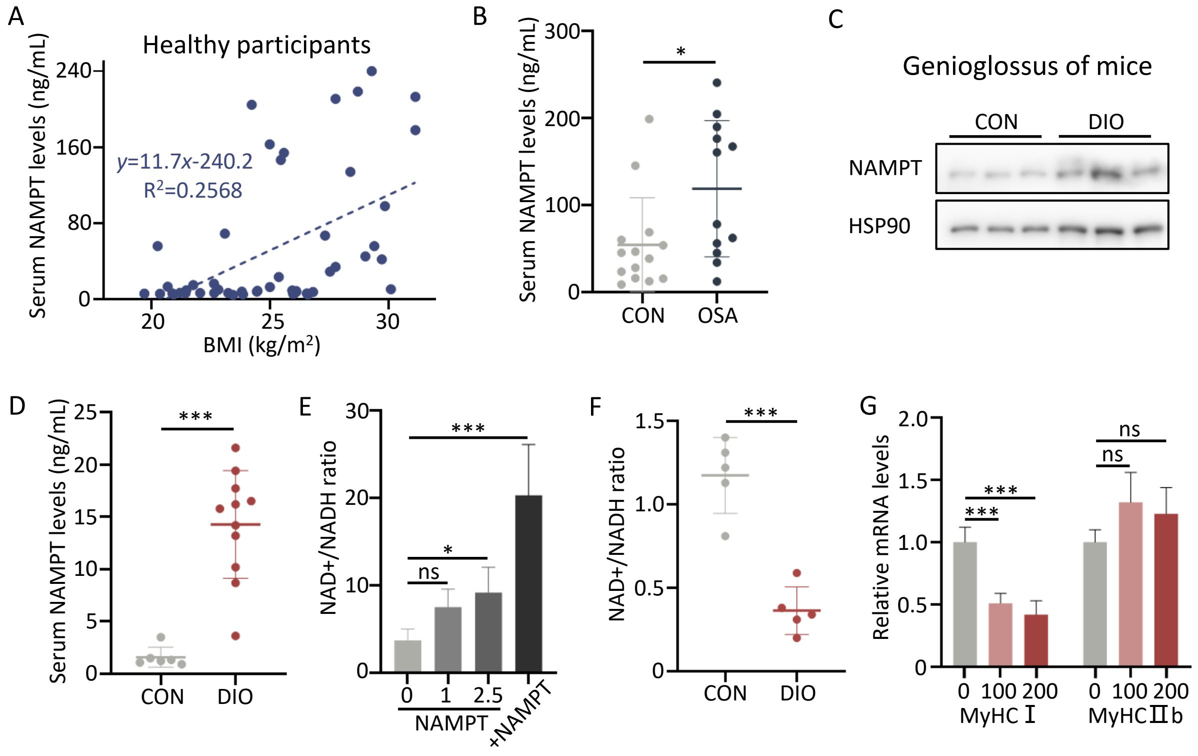

A
Healthy participants
Serum NAMPT levels (ng/mL)
y=11.7x-240.2
R2=0.2568
BMI (kg/m2)
B
Serum NAMPT levels (ng/mL)
CON
OSA
C
Genioglossus of mice
CON
DIO
NAMPT
HSP90
D
Serum NAMPT levels (ng/mL)
CON
DIO
E
NAD+/NADH ratio
NAMPT
+NAMPT
F
NAD+/NADH ratio
CON
DIO
G
Relative mRNA levels
MyHC Ⅰ
MyHCⅡb

Figure S3

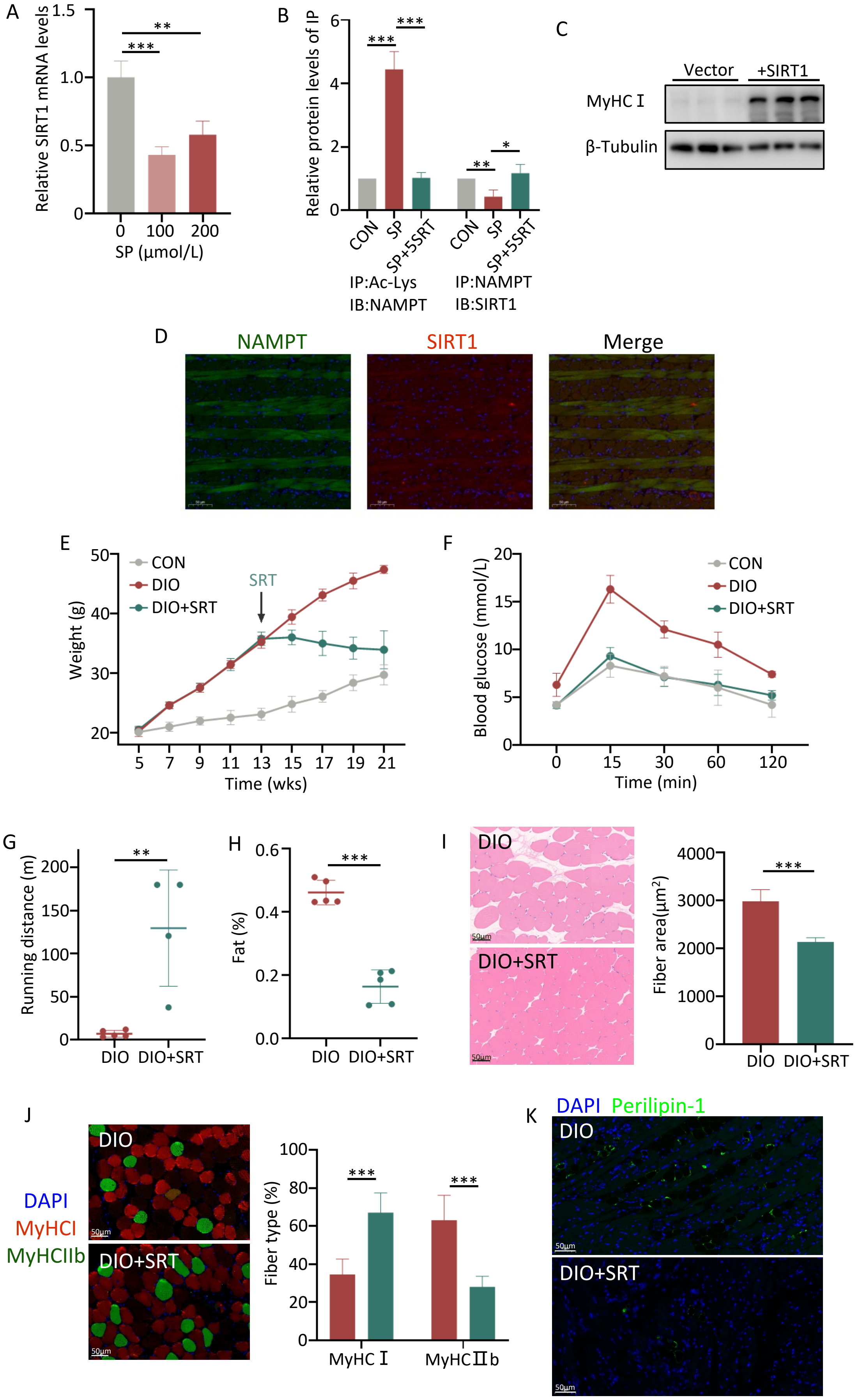
A
Relative SIRT1 mRNA levels
SP (μmol/L)
B
Relative protein levels of IP
CON
SP
SP+5SRT
IP:Ac-Lys
IB:NAMPT
IP:NAMPT
IB:SIRT1
C
Vector
+SIRT1
MyHC I
β-Tubulin
D
NAMPT
SIRT1
Merge
E
Weight (g)
CON
DIO
DIO+SRT
SRT
Time (wks)
F
Blood glucose (mmol/L)
Time (min)
G
Running distance (m)
H
Fat (%)
I
Fiber area(μm²)
J
DAPI
MyHCI
MyHCIIb
Fiber type (%)
MyHC I
MyHC IIb
K
DAPI Perilipin-1
50μm

Figure S4

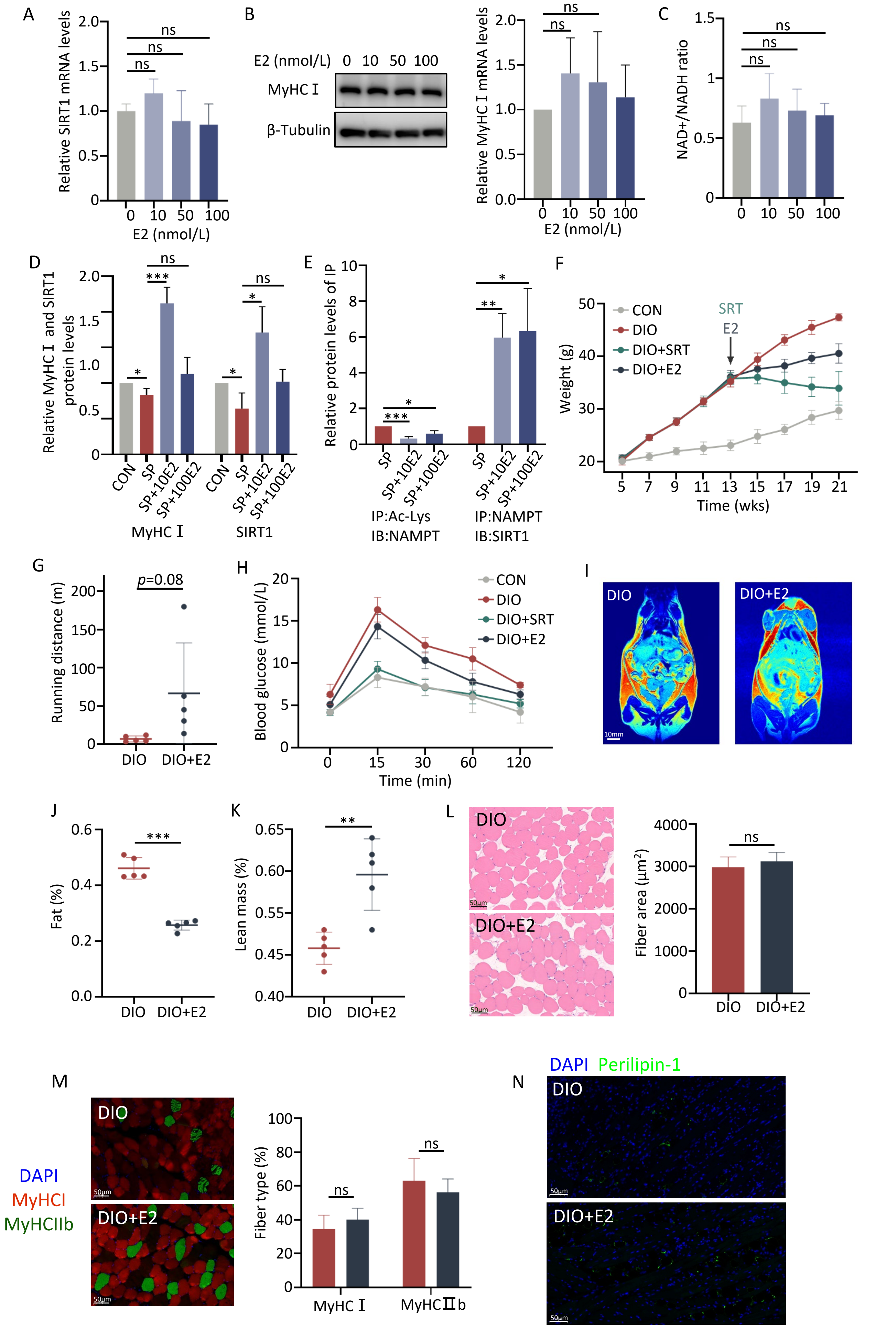
A
Relative SIRT1 mRNA levels
E2 (nmol/L)
ns
B
E2 (nmol/L) 0 10 50 100
MyHC I
β-Tubulin
Relative MyHC I mRNA levels
C
NAD+/NADH ratio
D
Relative MyHC I and SIRT1 protein levels
CON
SP
SP+10E2
SP+100E2
MyHC I
SIRT1
E
Relative protein levels of IP
IP:Ac-Lys
IB:NAMPT
IP:NAMPT
IB:SIRT1
F
Weight (g)
CON
DIO
DIO+SRT
DIO+E2
SRT
E2
Time (wks)
G
Running distance (m)
p=0.08
DIO
DIO+E2
H
Blood glucose (mmol/L)
Time (min)
I
DIO
DIO+E2
10mm
J
Fat (%)
K
Lean mass (%)
L
DIO
DIO+E2
50μm
Fiber area (μm²)
M
DAPI
MyHCI
MyHCIIb
Fiber type (%)
MyHC I
MyHC II b
N
DAPI Perilipin-1
