# Supplementary material for: Nicotinamide Phosphoribosyltransferase Acetylation Mediating Muscle Dysfunction Contributes to Sleep Apnoea in Obesity
Source: J Cachexia Sarcopenia Muscle. 2025 Feb 3;16(1):e13693. doi: 10.1002/jcsm.13693 (PMC11790607; doi:10.1002/jcsm.13693)
Supplement: Supplementary file 2 — Table S1. Baseline characteristics and body composition of the study population. [file JCSM-16-e13693-s002.docx]

**Nicotinamide Phosphoribosyltransferase Acetylation Mediating Muscle Dysfunction Contributes to Sleep Apnea in Obesity**

Journal of Cachexia, Sarcopenia and Muscle

Liu Zhang, Ya Ru Yan, Shi Qi Li, Ying Ni Lin, Yi Wang,Yu Qing Wang, Ning Li, Fang Ying Lu, Xian Wen Sun, Li Yue Zhang, Jian Ping Zhou, Yong Jie Ding, Qing Yun Li

Correspondence: Qing Yun Li, M.D, Ph.D.

Department of Respiratory and Critical Care Medicine, Ruijin Hospital, Shanghai Jiao Tong University School of Medicine, Shanghai 200025, China

Email: liqingyun68@hotmail.com

**Supplement Table S1** Baseline characteristics and body composition of the study population

|  |  | Mild or no OSA  n=40 | Moderate-to-severe OSA  n=70 | *p^#^* |
| --- | --- | --- | --- | --- |
| Age (y) |  | 41.9±15.8 | 50.34±12.1 | 0.002 |
| Male (n, %) |  | 27 (67.5%) | 56 (80%) | 0.143 |
| BMI (kg/m^2^) |  | 25.1±4.0 | 28.1±3.4 | <0.001 |
| AHI (times/h) |  | 7.2±4.1 | 32.4±12.3 | <0.001 |
| Triceps brachii | lean mass (%) | 38.04±12.6 | 38.08±15.1 | 0.989 |
|  | fat mass (%) | 31.73±10.5 | 27.4±9.8 | 0.03 |
| Rectus abdominis | lean mass (%) | 37.2±17.2 | 36.4±10.8 | 0.772 |
|  | fat mass (%) | 30.8±8.3 | 31.7±7.2 | 0.586 |
| Genioglossus | lean mass (%) | 68.2±11.4 | 59.8±11.6 | <0.001 |
|  | fat mass (%) | 18.8±5.2 | 21.7±6.0 | 0.014 |
| Total muscles | lean mass (%) | 49.42±15.1 | 45.0±10.9 | 0.111 |
|  | fat mass (%) | 33.1±8.0 | 34.2±6.2 | 0.416 |

Data are presented as mean±SD or n (%)

# t-test or χ2 test as appropriate.

Lean mass and fat mass were measured by electrical impedance mammography.

OSA, obstructive sleep apnea; BMI, body mass index; AHI, apnea-hypopnea index.
