# Supplementary material for: Nicotinamide Phosphoribosyltransferase Acetylation Mediating Muscle Dysfunction Contributes to Sleep Apnoea in Obesity
Source: J Cachexia Sarcopenia Muscle. 2025 Feb 3;16(1):e13693. doi: 10.1002/jcsm.13693 (PMC11790607; doi:10.1002/jcsm.13693)
Supplement: Supplementary file 4 — Data S1. Supplementary References. [file JCSM-16-e13693-s003.docx]

**Supplement reference**

S1. Chang, Y.-H.; Chang, D.-M.; Lin, K.-C.; Shin, S.-J.; Lee, Y.-J. Visfatin in Overweight/Obesity, Type 2 Diabetes Mellitus, Insulin Resistance, Metabolic Syndrome and Cardiovascular Diseases: A Meta-Analysis and Systemic Review. *Diabetes Metab. Res. Rev.* **2011**, *27*, 515–527.

S2. Gong, H.; Chen, H.; Xiao, P.; Huang, N.; Han, X.; Zhang, J.; Yang, Y.; Li, T.; Zhao, T.; Tai, H.; et al. miR-146a Impedes the Anti-Aging Effect of AMPK via NAMPT Suppression and NAD+/SIRT Inactivation. *Signal Transduct. Target. Ther.* **2022**, *7*, 66.

S3. Garratt, M.; Leander, D.; Pifer, K.; Bower, B.; Herrera, J.J.; Day, S.M.; Fiehn, O.; Brooks, S.V.; Miller, R.A. 17-α Estradiol Ameliorates Age-Associated Sarcopenia and Improves Late-Life Physical Function in Male Mice but Not in Females or Castrated Males. *Aging Cell* **2019**, *18*, e12920.

S4. Franklin, K.A.; Lindberg, E. Obstructive Sleep Apnea Is a Common Disorder in the Population-a Review on the Epidemiology of Sleep Apnea. *J. Thorac. Dis.* **2015**, *7*, 1311–1322.
